# Persistent HIV‐controllers are more prone to spontaneously clear HCV: a retrospective cohort study

**DOI:** 10.1002/jia2.25607

**Published:** 2020-09-09

**Authors:** Beatriz Dominguez‐Molina, Laura Tarancon‐Diez, Yusnelkis Milanés‐Guisado, Miguel Genebat, Salvador Resino, Carmen Rodriguez, Juan Gonzalez‐García, Norma Rallón, Maria Pernas, Concepción Casado, Cecilio Lopez‐Galíndez, Agathe León, Jose M Benito, Felipe García, Jorge Del Romero, Pompeyo Viciana, Luis F Lopez‐Cortes, Manuel Leal, Ezequiel Ruiz‐Mateos, ECRIS integrated in the Spanish AIDS Research Network

**Affiliations:** ^1^ Enfermedades Infecciosas Microbiología Clínica y Medicina Preventiva. Instituto de Biomedicina de Sevilla/Hospital Universitario Virgen del Rocío/CSIC/Universidad de Sevilla Sevilla Spain; ^2^ Laboratorio de Inmunovirologia Instituto de Biomedicina de Sevilla (IBiS), Hospital Universitario Virgen del Rocío/CSIC/Universidad de Sevilla Sevilla Spain; ^3^ Servicio de Urgencias Hospital Universitario Virgen del Rocío Sevilla Spain; ^4^ Unidad de Infección Viral e Inmunidad Centro Nacional de TOUCH FOR FMicrobiología Instituto de Salud Carlos III Madrid Spain; ^5^ Centro Sanitario Sandoval IdlSSC Madrid España; ^6^ Unidad de VIH, Hospital Universitario La Paz‐IdiPaz Madrid Spain; ^7^ Instituto de Investigación Sanitaria‐Fundación Jiménez Díaz Universidad Autónoma de Madrid (IIS‐FJD, UAM) Spain; ^8^ Hospital Universitario Rey Juan Carlos Móstoles Spain; ^9^ Unidad de Virologia Molecular Laboratorio de Referencia e Investigación en Retrovirus Centro Nacional de Microbiología Instituto de Salud Carlos III Madrid Spain; ^10^ Departmento de Enfermedades Infecciosas Hospital Clinic de Barcelona Hospital Clinic de Barcelona Barcelona Spain; ^11^ Servicio de Medicina Interna, Hospital Viamed Santa Ángela de la Cruz Seville Spain

**Keywords:** HIV, HCV, HIV‐controllers, persistent, transient, HCV spontaneous clearance

## Abstract

**Introduction:**

HIV‐controllers have the ability to spontaneously maintain viraemia at low or undetectable levels in the absence of antiretroviral treatment. Furthermore, HIV‐controllers seem to have a superior capacity to spontaneously clear hepatitis C virus (HCV) compared to non HIV‐controllers. Some of these subjects eventually lose HIV‐controller status (transient controllers), whereas some HIV‐controllers show a persistent natural HIV control (persistent controllers). We aimed to analyse whether persistent controllers have superior capacity to spontaneously clear HCV compared to transient controllers.

**Methods:**

We recruited HIV‐controllers from January 1981 up to October 2016 with available antibodies to HCV (anti‐HCV) data (n = 744). Factors associated with HIV spontaneous control in relation to HCV status were analysed in persistent and transient HIV‐controllers with anti‐HCV positive (n = 202 and n = 138 respectively) in comparison with 1700 HCV positive non HIV‐controllers recruited from January 1981 up to March 2018, bivariate and multivariate analyses, following a logistic regression model, were applied. In addition, the factors related to the loss and time to lose HIV‐controller status were explored (n = 744) using Log rank test and Kaplan–Meier curves, in this case the multivariate analysis consisted in a Cox regression model.

**Results:**

A higher frequency of HCV spontaneous clearance was found in persistent HIV‐controllers (25.5%) compared to non‐controllers (10.2%). After adjusting for potential confounders, as sex, age, HIV transmission risk, CD4^+^ T‐cell nadir and time of follow‐up, HCV clearance was independently associated with persistent HIV spontaneous control (*p* = 0.002; OR (95% CI) = 2.573 (1.428 to 4.633)), but not with transient spontaneous control (*p* = 0.119; 1.589 (0.888 to 2.845)). Furthermore, persistent HIV‐controllers were more likely to spontaneously clear the HCV in comparison with transient controllers (*p* = 0.027; 0.377 (0.159 to 0.893). Finally, not to lose or lengthen the time of losing this control was independently associated with HCV spontaneous clearance (*p* = 0.010; 0.503 (0.297 to 0.850).

**Conclusions:**

This study shows an association between spontaneous persistent HIV‐control and HCV spontaneous clearance. The study findings support the idea of preserved immune mechanisms in persistent HIV control implicated in HCV spontaneous clearance. These results highlight persistent HIV‐controllers but not transient controllers as a good model of functional HIV cure.

## 1 INTRODUCTION

HIV‐controllers are a small group of subjects (<1%) that maintain low (viraemic controllers) or undetectable (elite controllers) HIV viral load (VL) in the absence of antiretroviral treatment [1]. In large cohort studies [2, 3], it is clear that this phenotype is heterogeneous with some HIV‐controllers eventually losing the spontaneous viral control capacity, these individuals are named transient controllers as opposed to persistent controllers [4]. Understanding the mechanisms involved in this phenomenon is quite important. On one hand factors associated with the loss of HIV‐controller status may assist giving an optimum clinical care to these individuals and reciprocally, persistent controllers may serve as a good model for functional HIV cure. The mechanisms and attributes of this extraordinary phenotype of exceptionally long‐term control are still not completely understood [5, 6, 7]. Recent studies show that persistent HIV‐controllers in comparison with transient controllers have higher levels of HIV‐specific T‐cell response, low viral evolution, low levels of inflammation and a peculiar proteomic and metabolomic profile [4, 8, 9].

On the other hand, HIV‐controllers as a whole seem to have a higher capacity to spontaneously clear hepatitis C virus (HCV) compared to non HIV‐controllers [10, 11], although other studies have not found this enhanced capacity [12, 13]. Besides, it remains unknown whether persistent HIV‐controllers have a higher capacity to spontaneously clear HCV. According to the improved immunological capacities mentioned earlier, we hypothesize that persistent HIV‐controllers may have a better ability to spontaneously clear HCV. These findings could lead to significant advances on the chronic viral infections therapeutically designs as they act as an additional attribute of the persistent HIV‐controllers as a model for a HIV functional cure.

In this study we have been able to investigate HCV status profile in the large cohort of HIV‐controllers of the Spanish AIDS Research Network (ECRIS). Thus, the aim of this study was to analyse whether persistent HIV‐controllers had superior capacity to spontaneously clear HCV compared to transient HIV‐controllers.

## 2 METHODS

### 2.1 Patients and variables

Patients’ clinical and epidemiological data were included in the cohort of HIV‐controllers of the AIDS Research Network (ECRIS). This is an open, multicentre cohort of HIV controllers whose data come from the Long‐Term Non‐Progressors cohort and the Cohort of the Spanish AIDS Research Network (CoRIS) [14, 15] and other clinical centres across Spain. This cohort has been previously published [3, 16]. See Annex S1 for participating centres. In the last update, October 2016, this cohort included 807 HIV‐controllers.

This is a retrospective cohort study. Inclusion criteria were patients with VL < 2000 HIV RNA copies/mL ((elite controllers, VLs below the detection limit [<50 HIV‐RNA copies/mL], n = 273) and viraemic controllers (controllers with VLs between 50 and 2000 HIV‐RNA copies/mL, n = 471)) in the absence of antiretroviral treatment for at least one year of follow‐up and with available antibodies to HCV (anti‐HCV) data (n = 744). HIV‐controllers were split into “Transient HIV‐controllers” (n = 333), subjects who lost HIV‐controller status defined as loss of virological control (first viral measurement of a sustained increase of >2000 HIV RNA copies/mL during more than one year of follow‐up) or initiation of an antiretroviral treatment, as previously reported [16]. The same criteria were applied for the loss of elite controller status but with a sustained increase of >50 HIV RNA copies/mL during more than one year. Subjects without loss of HIV‐controller status were defined as “Persistent HIV‐controllers” (n = 411). HCV spontaneous clearers were defined as patients exposed to HCV (anti‐HCV positive) with qualitative negative HCV PCR in the absence of HCV specific treatment. These three data (anti‐HCV positive, HCV PCR and HCV specific treatment) must be available to classify a patient as a HCV spontaneous clearer.

For the analysis of the factors associated with persistent and transient HIV spontaneous control based on HCV profile, we selected HCV exposed persistent (n = 202) and transient (n = 138) HIV‐controllers and non HIV‐controllers (n = 1700). HCV exposed HIV non‐controllers were subjects with consecutive visits to the Infectious Disease Unit at Virgen del Rocío University Hospital (Seville, Spain) from January 1981 up to March 2018. These subjects were administered ART based on clinical criteria of contemporary guidelines.

For the analysis of factors associated with the loss of HIV spontaneous control we used the complete HIV ECRIS cohort (n = 744) with available anti‐HCV data. In addition, we performed the analysis of variables associated with the time to lose HIV‐controller status. The time to lose HIV‐controller status was considered the time period between the HIV diagnosis and the loss of HIV‐controller status. The subjects who did not lose HIV‐controller status were censored in the last follow‐up point.

All subjects gave written informed consent in accordance with the Declaration of Helsinki. The protocol was approved by the Comité de Etica de la Investigación at the Hospital Universitario Virgen del Rocío de Sevilla (2012PI/240).

Routinely, the absolute numbers of CD4^+^ T cells were assayed from fresh whole blood samples, using the Epics XL‐MCL flow cytometer (Beckman Coulter). Plasma HIV RNA load was measured in fresh samples by quantitative polymerase chain reaction (PCR; COBAS Ampliprep/COBAS Taqman HIV test; Roche molecular systems), according to the manufacturer’s instructions. The detection limit was 50 HIV RNA copies/mL. Qualitative and quantitative reverse transcription PCR was performed for plasma HCV RNA amplification (COBAS Amplicor; Roche Diagnosis, Barcelona, Spain), with detection limit of 15 IU/mL. HCV genotype was determined using a reverse‐hybridization assay (InnoLIPA HCV II; Innogenetics, Barcelona, Spain).

### 2.2 Statistical analysis and Covariates

Continuous variables were expressed as median and interquartile range (IQR) and categorical variables were expressed as number and percentage. Bivariate and multivariate analyses, following a logistic regression model, were applied for the analysis of factors associated with persistent and transient HIV spontaneous control and for the analysis of factors associated with loss of HIV‐controller status. For these two analyses the dependent variables were being HIV‐controller (persistent and transient) and loss of HIV‐control respectively. For the analysis of time to lose HIV‐controller status, the time to lose this condition was considered the dependent variable, Log rank test and Kaplan–Meier curves were used. In this case the multivariate analysis consisted in a Cox regression model, using the enter procedure. As covariates these models were adjusted by sex, age at HIV diagnosis, time of follow‐up, people who inject drugs as (PWID) as risk of HIV transmission, CD4^+^ T‐cell nadir, HLA‐B*57 typing, and finally by HIV elite‐controller status and HCV spontaneous clearance (as describe earlier), as potential confounders. All variables with a *p* < 0.1 were included in the multivariate analysis, and *p* < 0.05 was considered statistically significant. Statistical analyses were performed using SPSS software version 22 (IBM SPSS, Chicago, Illinois) and graphs were generated with Prism, version 6.0 (GraphPad Software, Inc.).

## 3 RESULTS

### 3.1 Higher frequency of HCV spontaneous clearance in persistent HIV‐controllers, but not in transient HIV‐controllers, compared to non‐controllers

First, we found that the rate of HCV spontaneous clearance in the overall HIV‐controller population (n = 340) was higher compared with non‐controllers (n = 1700) (19.4% vs. 10.2% respectively) confirming previous results in small cohorts [10, 11]. After adjusting for sex, age at HIV diagnosis, time of follow‐up and the risk of transmission — frequency of PWID —, as potential confounders, HCV spontaneous clearance remained independently associated with HIV spontaneous control (p < 0.001; 2.180 (1.472 to 3.229). Afterwards, we compared HCV‐exposed persistent (n = 202) and transient HIV‐controllers (n = 138) with the 1700 HCV positive HIV non‐controllers (Table 1). We found that in both groups there was a higher proportion of women, higher nadir CD4^+^ T‐cell levels, lower frequency of patients at clinical stage CDC C, a higher representation of HCV genotype 3 and a lower representation of genotype 1 and, as expected, an overrepresentation of HLA‐B57 + subjects compared to HIV‐non‐controllers (Table 1). However, persistent HIV‐controllers presented higher rates of HCV spontaneous clearance than HIV non‐controllers (25.5% vs. 10.2%), although this was not the case for transient HIV‐controllers (14.5% vs. 10.2%). After adjusting for potential confounders, HCV spontaneous clearance remained independently associated with persistent HIV spontaneous control but not with transient (Table 2). Due to the scarce data, we could not adjust for HLA‐B*57 typing, but it was associated with both persistent and transient control (Table 2).

**Table 1:** Characteristics of persistent and transient HIV‐controllers and HIV/HCV positive non‐controllers

	Persistent HIV‐controllers (n = 202)	Transients HIV‐controllers (n = 138)	HIV non‐controllers (n = 1700)	Persistent HIV‐controllers vs. HIV non‐controllers	Transient HIV‐controllers vs. HIV non‐controllers
				*p*; OR (CI 95%)	*p*; OR (CI95%)
Female sex (n) %	66/202 (32.7)	49/138 (35.5)	235/1700 (13.8)	**<0.001; 3.025 (2.187 to 4.186)**	**<0.001; 3.432 (2.359 to 4.993)**
Age at HIV diagnosis	28 [24 to 33]	28 [23 to 33]	27 [23 to 33]	0.360; 1.008 (0.991 to 1.025)	0.667; 1.004 (0.984 to 1.025)
HIV transmission risk, PWID (n) %	162/202 (80.2)	102/138 (73.9)	1379/1700 (83.6)	0.226; 0.796 (0.550 to 1.152)	**0.004; 0.557 (0.373 to 0.832)**
PWID, female (n) %	47/66 (71.2)	32/49 (65.3)	161/235 (70.9)	0.964; 1.074 (0.554 to 1.857)	0.437; 0.772 (0.401 to 1.484)
CD4 nadir	520 [388 to 675]	285 [213 to 396]	111 [31 to 224]	**<0.001; 1.009 (1.008 to 1.010)**	**<0.001; 1.006 (1.005 to 1.007)**
Time of follow‐up (years)^a^	18.1 [11.7 to 23.7]	15.8 [8.0 to 21.0]	18.6 [11.8 to 24.9]	0.578; 0.995 (0.978 to 1.012)	**<0.001; 0.961 (0.942 to 0.981)**
Clinical stage C (n) %^b^	7/107 (6.5)	10/121 (8.3)	275/1674 (16.4)	**0.009; 0.356 (0.164 to 0.775)**	**0.020; 0.458 (0.237 to 0.887)**
HCV PCR+ (n) %^c^	99/123 (80.5)	113/130 (86.9)	1521/1694 (89.8)	**0.002; 0.469 (0.292 to 0.753)**	0.304; 0.756 (0.443 to 1.289)
HCV genotype detected (n) %^d^					
Genotype 1	28/132 (40.0)	37/84 (44.0)	616/1075 (57.3)	**0.005; 0.497 (0.303 to 0.813)**	**0.019; 0.587 (0.375 to 0.918)**
Genotype 2	2/132 (2.9)	3/84 (3.6)	9/1075 (0.8)	0.115; 3.484 (0.738 to 16.440)	**0.029; 4.387 (1.165 to 16.521)**
Genotype 3	23/132 (32.9)	31/84 (36.9)	237/1075 (22)	**0.038; 1.730 (1.030 to 2.908)**	**0.002; 2.068 (1.298 to 3.296)**
Genotype 4	17/132 (24.2)	13/84 (15.5)	213/1075 (19.8)	0.367; 1.298 (0.737 to 2.287)	0.335; 0.741 (0.403 to 1.364)
HCV spontaneous clearers (n) %^e^	24/94 (25.5)	17/117 (14.5)	173/1694 (10.2)	**<0.001; 3.014 (1.847 to 4.918)**	0.143; 1.495 (0.873 to 2.559)
HLA‐B*57 (n) %^f^	25/79 (31.6)	12/56 (21.4)	41/818 (5)	**<0.001; 8.774 (4.968 to 15.45)**	**<0.001; 5.169 (2.538 to 10.53)**

*p* values < 0.05 are shown in bold. CI, confidence interval;.OR, odd ratio; PWID, people who inject drug.

^a^ Time period between HIV diagnosis and loss of HIV‐controller status or last monitored visit point

^b^ clinical stage C data from 107 persistent HIV‐controllers, 121 transient HIV‐controllers and 1674 non‐controllers

^c^ HCV PCR + was considered before HCV treatment onset in those who were treated

^d^ HCV genotype detected from 132 persistent HIV‐controllers, 84 transient HIV‐controllers and 1075 non‐controllers

^e^ for HCV spontaneous clearance calculation we only considered patients with available data of anti‐HCV, HCV PCR and HCV treatment. 94 persistent HIV‐controllers, 117 transient HIV‐controllers and 1694 non‐controllers accomplished all data.

^f^ HLA‐B*57 data from 79 persistent HIV‐controllers, 56 transient HIV‐controllers and 818 non‐controllers.

**Table 2:** Factors associated with persistent and transient HIV spontaneous control

Variables	Persistent HIV‐control	Transient HIV‐control
	Adjusted *p*; OR (IC 95%)	Adjusted *p*; OR (IC 95%)
HCV spontaneous clearance	**0.002; 2.573 (1.428 to 4.633)**	0.119; 1.589 (0.888 to 2.845)
Female sex	**0.006; 2.176 (1.250 to 3.790)**	**<0.001; 2.854 (1.823 to 4.467)**
CD4 nadir	**<0.001; 1.009 (1.008 to 1.010)**	**<0.001; 1.006 (1.005 to 1.007)**
Time of follow‐up	NA	**0.002; 0.961 (0.938 to 0.985)**
HIV transmission risk, PWID	NA	0.530; 0.855 (0.523 to 1.396)

*p* values < 0.05 are shown in bold. CI, confidence interval; NA, not applicable; OR, odd ratio, PWID, people who inject drug.

When we restricted the analysis to persistent elite controllers (n = 143) and transient elite controllers (n = 43) (Table 3), again a higher frequency of HCV spontaneous clearers was found in persistent elite controllers than in non‐controllers (20.6% vs. 10.2%) but not in transient elite controllers ((11.1% vs. 10.2%) (*p* = 0.008; OR (95%) = 2.279 (1.240 to 4.189) and *p* = 0.860; OR (95%) = 1.099 (0.384 to 3.144) respectively). In the multivariate analysis, in a first model after adjusting by sex, again, HCV spontaneous clearance was associated with persistent elite HIV control (*p* = 0.016; OR (95%) = 2.127 (1.149 to 3.938). In a second model after adjusting by sex and CD4^+^ T‐cell nadir levels this correlation was lost (*p* = 0.123; OR (95%) = 1.749 (0.859 to 3.558).

**Table 3:** Characteristics of persistent and transient HIV elite‐controllers and HIV/HCV positive non‐controllers

	Persistent elite controllers (n = 143)	Transients elite controllers (n = 43)	HIV non‐controllers (n = 1700)	Persistent HIV‐controllers vs. HIV non‐controllers	Transient HIV‐controllers vs. HIV non‐controllers
				*p*; OR (CI95%)	*p*; OR (CI95%)
Female sex (n) %	50/143 (35.0)	17/43 (39.5)	235/1700 (13.8)	**<0.001; 3.352 (2.314 to 4.854)**	**<0.001; 4.076 (2.178 to 7.628)**
Age at HIV diagnosis	28 [23 to 33]	29 [24 to 35]	27 [23 to 33]	0.397; 1.009 (0.989 to 1.029)	0.080; 1.028 (0.997 to 1.060)
HIV transmission risk, PWID (n) %	115/143 (80.4)	32/43 (74.4)	1379/1700 (83.6)	0.332; 0.807 (0.523 to 1.245)	0.116; 0.572 (0.285 to 1.148)
PWID, female (n) %	47/66 (71.2)	32/49 (65.3)	161/235 (70.9)	0.964; 1.074 (0.554 to 1.857)	0.437; 0.772 (0.401 to 1.484)
Time of follow‐up (years)	18.5 [13.0 to 24.7]	17.4 [8.1 to 22.6]	18.6 [11.8 to 24.9]	0.491; 1.007 (0.987 to 1.028)	**0.094; 0.971 (0.938 to 1.005)**
CD4 nadir	510 [363 to 680]	292 [220 to 420]	111 [31 to 224]	**<0.001; 1.009 (1.008 to 1.011)**	**<0.001; 1.005 (1.004 to 1.007)**
Clinical stage C (n) %^a^	7/72 (9.7)	3/41 (7.0)	275/1674 (16.4)	0.136; 0.548 (0.249 to 1.208)	0.131; 0.402 (0.123 to 1.310)
HCV PCR+ (n) %^b^	76/90 (84.4)	38/42 (90.5)	1521/1694 (89.8)	0.110; 0.617 (0.342 to 1.115)	0.884; 1.081 (0.381 to 3.064)
HCV genotype detected (n) %^c^					
Genotype 1	23/54 (42.6)	11/28 (39.3)	616/1075 (57.3)	**0.036; 0.553 (0.318 to 0.961)**	**0.063; 0.482 (0.224 to 1.039)**
Genotype 2	1/54 (1.9)	0/28 (0)	9/1075 (0.8)	0.450; 2.235 (0.278 to 17.956)	0.999
Genotype 3	16/54 (29.6)	13/28 (46.4)	237/1075 (22)	0.195; 1.489 (0.816 to 2.717)	**0.004; 3.064 (1.438 to 6.530)**
Genotype 4	14/54 (25.9)	4/28 (14.3)	213/1075 (19.8)	0.276; 1.416 (0.757 to 2.651)	0.470; 0.674 (0.232 to 1.965)
HCV spontaneous clearers (n) %^d^	14/68 (20.6)	4/36 (11.1)	173/1694 (10.2)	**0.008; 2.279 (1.240** to **4.189)**	0.860; 1.099 (0.384 to 3.144)
HLA‐B*57 (n) %^e^	25/64 (39.1)	3/20 (15.0)	41/818 (5)	**<0.001; 12.15 (6.72 to 22.00)**	**0.062; 3.344 (0.942 to 11.87)**

*p* values < 0.1 are shown in bold. CI, confidence interval; NA, not applicable; OR, odd ratio; PWID, people who inject drug.

^a^ Clinical stage C data from 72 persistent HIV‐controllers, 41 transient HIV‐controllers and 1674 non‐controllers

^b^ HCV PCR+ was considered before HCV treatment onset in those who were treated

^c^ HCV genotype detected from 54 persistent HIV‐controllers, 28 transient HIV‐controllers and 1075 non‐controllers.

### 3.2 HCV spontaneous clearance was independently associated with persistent HIV‐controller status

We analysed the whole HIV‐controller subset in detail (n = 744). Table 4 shows the differences between persistent controllers and transient controllers. Persistent controllers had longer time of follow‐up, higher CD4 T‐cell nadir and higher frequency of elite control, PWID and HLA‐B*57 carriers than transient controllers. We also categorized patients based on the calendar year of HIV diagnosis. Interestingly, while there were more persistent controllers HIV‐diagnosed in the 80s decade, there were more transient controllers HIV‐diagnosed in the 2000s. It is important to note that there were no differences in HCV genotype distribution between groups (Table 4). These results point out that the loss of control was not associated with the different genotype distribution.

**Table 4:** Characteristics of HIV‐controllers stratified according to the loss of HIV control status

	Persistent controllers (411)	Transient controllers (n = 333)	*p*; OR (IC 95%)
Female sex (n) %	114/417 (27.7)	111/333 (33.3)	**0.099; 1.303 (0.952 to 1.783)**
Age at HIV diagnosis	29 [24 to 35]	30 [25 to 36]	0.334; 1.008 (0.992 to 1.024)
Calendar year of HIV diagnosis			
1980 to 1989	91/411 (22.1)	52/332 (15.7)	**0.027; 0.653 (0.448 to 0.952)**
1990 to 1999	99/411 (24.1)	94/332 (28.3)	0.192; 1.245 (0.896 to 1.729)
2000 to 2009	157/411 (38.2)	174/332 (52.4)	**<0.001; 1.782 (1.329 to 2.389)**
2010 to 2015	64/411 (15.6)	12/332 (3.6)	**<0.001; 0.203 (0.108 to 0.384)**
Time of follow‐up (years)^a^	9.4 [4.1 to 19.1]	5.7 [3.0 to 14.7]	**<0.001; 0.960 (0.942 to 0.977)**
HIV transmission risk, PWID (n) %	167/411 (40.6)	106/333 (31.8)	**0.013; 0.682 (0.504 to 0.924)**
CD4 T‐cell nadir	550 [419 to 710]	327 [253 to 443]	**<0.001; 0.994 (0.993 to 0.995)**
Clinical stage C (n) %^b^	7/296 (2.4)	12/305 (3.9)	0.277; 1.691 (0.656 to 4.356)
Elite HIV‐controller status (n) %	207/411 (50.4)	66/333 (19.8)	**<0.001; 0.244 (0.175 to 0.339)**
Anti‐HCV+ (n, %)	202/411 (49.1)	138/333 (41.4)	**0.036; 0.732 (0.547 to 0.980)**
HCV PCR+ (n,%)^c^	99/332 (29.8)	113/325 (34.8)	0.175; 1.254 (0.904 to 1.741)
Log_10_ HCV VL (IU/mL)^d^	5.85 [5.40 to 6.40]	6.07 [5.41 to 6.60]	0.289; 1.166 [0.878 to 1.550]
HCV genotype detected (n) %^e^			
Genotype 1	28/70 (40)	37/84 (44)	0.613; 1.181 (0.620 to 2.248)
Genotype 2	2/70 (2.9)	3/84 (3.6)	0.804; 1.259 (0.204 to 7.756)
Genotype 3	23/70 (32.9)	31/84 (36.9)	0.600; 1.195 (0.613 to 2.329)
Genotype 4	17/70 (24.3)	13/84 (15.5)	0.172; 0.571 (0.255 to 1.277)
HCV clearance (n%)^f^	24/94 (25.5)	17/117 (14.5)	**0.047; 0.496 (0.248 to 0.991)**
HLA‐B*57 (n) %^g^	31/105 (29.5)	15/96 (15.6)	**0.021; 0.442 (0.221 to 0.883)**

*p* values < 0.1 are shown in bold. CI, confidence interval; NA, not applicable; OR, odd ratio; PWID, people who inject drug.

^a^ Time period between HIV diagnosis and loss of HIV‐controller status or last monitored visit point

^b^ clinical stage C data from 296 persistent controllers, and 305 transient controllers

^c^ HCV PCR data available for 332 persistent controllers and 325 transient controllers. HCV PCR+ was considered before HCV treatment onset in those who were treated

^d^ HCV VL data from 96 persistent controllers and 101 transient controllers

^e^ HCV genotype detected in 70 persistent controllers, and 84 transient controllers

^f^ for HCV spontaneous clearance calculation we only considered patients with anti‐HCV+ and available data of HCV PCR and HCV treatment. HCV spontaneous clearance was calculated in 94 persistent controllers and 117 transient controllers

^g^ HLA‐B*57 data from 105 persistent controllers, and 96 transient controllers.

Regarding HCV profile, HIV persistent controllers had higher frequency of anti‐HCV positive than transient controllers. Interestingly, there was a higher proportion of HCV spontaneous clearers in persistent controllers. In a multivariate analysis, the factors independently associated with loss of HIV‐controller status were lower CD4 T‐cell nadir, non‐elite controller status and not achieving HCV spontaneous clearance (table 5). We also adjusted by calendar year of HIV positive instead of by the time of follow‐up, as these were co‐linear variables and similar results were found (data not shown). We could not adjust simultaneously by HLA‐B*57 and spontaneous HCV clearance due to the low number of subjects sharing both determinations. However, when we included HLA‐B*57 genotype in the previous model no association was found between this variable and the loss of HIV‐controller status (*p* = 0.384; OR (95%) = 0.650 (0.247 to 1.714)).

**Table 5:** Factors associated with loss of HIV‐controller status

	Adjusted *p*; OR (IC 95%)
Female sex	0.373; 1.382 (0.679 to 2.815)
Time of follow‐up	0.328; 0.978 (0.936 to 1.022)
HIV transmission risk, PWID	0.149;1.767 (0.816 to 3.827)
CD4 T‐cell nadir	**<0.001; 0.995 (0.993 to 0.997)**
Elite HIV‐controller status	**<0.001; 0.136 (0.068 to 0.274)**
HCV spontaneous clearance	**0.027; 0.377 (0.159 to 0.893)**

*p* values < 0.05 are shown in bold. CI, confidence interval; OR, odd ratio; PWID, people who inject drug.

The same results were obtained when the patients were categorized based only on the loss of virological control, not on the loss of HIV controller status (n = 563 and 181 respectively). After adjusting by sex, time of follow‐up and PWID, again: CD4 T‐cell nadir, the frequency of elite controllers and HCV spontaneous clearance were independently associated with not having loss virological control ((*p* < 0.001; OR (95%) = 0.995 (0.993 to 0.997), *p* < 0.001; OR (95%) = 0.136 (0.068 to 0.274) and *p* = 0.027; OR (95%) = 0.377 (0.159 to 0.893) respectively)).

### 3.3 HCV spontaneous clearance was associated with the time to maintain HIV‐controller status

**Figure 1:**
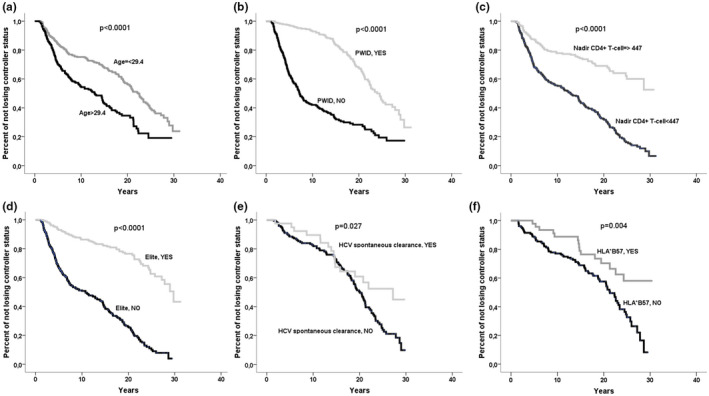
Kaplan–Meier curves for time to loose HIV‐controller status analysis. Percent of subjects not losing HIV controller status are shown for **(A)** Age dichotomized using the median (29.4 [24.6–35.4]) as the cut‐off value, **(B)** people who inject drugs (PWID), **(C)** Nadir CD4^+^ T‐cell counts dichotomized using the median (447 [304–622]) as the cut‐off value, **(D)** elite controller, **(E)** HCV spontaneous clearance and **(F)** carriers of HLA*B57. Bivariate analysis using a Log‐rank test was performed to assess significant differences among the curves.

Another way of analysing the results is to perform a time to event analysis. The event was the loss of HIV‐controller status. The median time of follow‐up was 7.2 [3.7 to 17.7] years. The analysis revealed that younger age at diagnosis, PWID as risk factor, high CD4^+^ T‐cell nadir, being elite, HLA‐B*57 carriers and having cleared HCV spontaneously were associated with maintaining HIV‐controller status overtime (Figure 1). After adjusting by all these variables, but HLA‐B*57, in a Cox regression model, having cleared HCV spontaneously was independently associated with the time to maintain HIV‐controller status together with the rest of the variables mentioned earlier (Table 6). However, when we included HLA‐B*57 genotype in the previous model no association was found between this variable and the time to lose HIV‐controller status (*p* = 0.229; OR (95%) = 0.703 (0.396 to 1.249)).

**Table 6:** Factors associated with the time to lose HIV‐controller status

	Univariate *p*; OR (IC 95%)	Adjusted *p*; OR (IC 95%)
Female sex (n) %	0.831; 1.025 (0.816 to 1.288)	
Age at HIV diagnosis >29.4 years^a^	**<0.001; 1.929 (1.541 to 2.416)**	**<0.001; 2.301 (1.545 to 3.427)**
HIV transmission risk, PWID (n) %	**<0.001; 0.262 (0.206 to 0.334)**	**0.016; 0.587 (0.380 to 0.906)**
CD4 T‐cell nadir >447 cells/mm^a^	**<0.001; 0.337 (0.262 to 0.432)**	**0.003; 0.478 (0.296 to 0.772)**
Elite HIV‐controller status (n) %	**<0.001; 0.199 (0.151 to 0.263)**	**<0.001; 0.264 (0.172 to 0.406)**
HCV spontaneous clearance (n) %	**0.029; 0.562 (0.335 to 0.943)**	**0.010; 0.503 (0.297 to 0.850)**
HLA‐B*57 (n) %	**0.004; 0.446 (0.256 to 0.779)**	

*p* values < 0.05 are shown in bold. CI, confidence interval; OR, odd ratio; PWID, people who inject drug.

^a^ Patients were categorized according to the median age at diagnosis; 29.4 [24.6 to 35.4]

^b^ patients were categorized according to the median CD4^+^ T‐cell nadir; 447 [304 to 622].

## 4 DISCUSSION

Results presented herein show that persistent HIV‐controllers, as opposed to what happened to transient HIV‐controllers, had a better ability to spontaneously clear HCV. In fact, the time to lose HIV‐controller status was associated with HCV spontaneous clearance.

HIV‐controller is a heterogeneous phenotype [2, 3], some HIV‐controllers eventually lose HIV‐controller status [17, 18, 19]. It is a key to detect these patients before the loss of control takes place and differentiate them of the persistent HIV‐controllers. This will enable a correct treatment in transient HIV‐controllers to avoid non AIDS events, as cardiovascular diseases [16, 20, 21] and, on the other hand, the persistent control phenotype will become a good model of persistent HIV‐remission in order to design immunotherapeutic strategies [7]. In this manuscript we describe a new characteristic of persistent HIV‐controllers as is a higher ability to spontaneously clear HCV unlike transient controllers. Cohort studies are needed to validate biomarkers associated with the loss of HIV control, independently of HCV clearance status, to offer these subjects therapeutic options, not necessarily cART [22].

HIV persistent controllers have been recently characterized as having low levels of proviral diversity, low reservoir size and high levels of HIV‐specific T‐cell polyfunctionality [4] associated with a specific proteomic and metabolomic profile [8, 9]. Many of these subjects were PWID and had multiple re‐exposures to HCV during the first years of being positive that mainly occurred in the 80s and early 90s and anti‐VHC and HCV RNA measurements were later available, if after all this re‐exposures the individual remained with negative ARN for HCV tell us about the preserved ability of those subjects to spontaneously clear the virus. We believe that independently the individuals became positive with one virus before the other, normally HCV precedes HIV positivity and the potential re‐exposures to HCV, it will be interesting to extent immunological correlates [23] of persistent HIV control and HCV spontaneous clearance to deeply dissect the intrinsic host mechanisms involved in this extraordinary phenotype. We have recently found that these subjects, able to clear HCV and spontaneously control HIV, referred as “supercontrollers,” showed higher levels of HCV‐specific CD4^+^ T‐cell polyfunctionality, higher levels of CD8 + CD161 T‐cells together with low T‐cell exhaustion associated with preserved innate immunity features [23]. This sustained immunity is also reflected in the rates of HCV spontaneous clearance in these subjects (25.5%) which is similar to that found in HCV monoinfected patients in Spain (22%) [24]. Hence, supercontrollers, who are enriched in persistent controllers, contribute with these mechanisms of sustained immunity to very long‐term persistent HIV remission.

Based on this study, the higher rates of HCV spontaneous clearance among HIV‐controllers, found previously in small cohorts [10, 11], were mainly due to the contribution of persistent but not transient HIV‐controllers. This was true even after adjusting by elite controller status subset that was enriched in persistent controllers and when considering the loss of virological control instead of the loss of HIV‐controller status. As expected, we had an overrepresentation of HLA‐B*57 carriers among HIV‐controllers, which has been extensively reported [25, 26, 27]. Some authors have previously observed higher HCV clearance among patients who carried HLA‐B*57 alleles [28]. Nevertheless, we did not find an association of HLA‐B*57 with HCV spontaneous clearance neither in HIV‐controllers (persistent or transient, *p* = 0.2; 2.6 [0.6 to 11.8] and *p* = 0.6; 1.6 [0.3 to 7.4] respectively) nor HIV non‐controllers separately (*p* = 0.3; 1.6 [0.7 to 3.6]). Asher et al., have communicated that other factors besides HLA‐B*57, such as HIV spontaneous control, were associated with HCV spontaneous clearance [29] in agreement with our results.

In the present study we found no differences in HCV genotype distribution between persistent and transient HIV‐controllers, while we found a different HCV genotype distribution in the overall HIV‐controller population vs. non‐controllers [12]. Therefore, the most plausible explanation for this fact is that HIV‐controllers spontaneously cleared genotype 1 more easily than non‐controllers, as it occurs in general population [30]. Unfortunately we did not have enough subjects in acute HCV infection in order to know HCV‐genotype preferential clearance in HIV‐controllers.

This study describes a new attribute of persistent HIV‐controllers, which is the enhanced capacity in people living with HIV to clear other chronic viral infection as HCV. Persistent controllers, that have special characteristics, in the case of supercontrollers, consisting on an enhance T‐cell response [23], a specific proteomic and metabolomic profile [8, 9], low levels of inflammation [4] and very low viral diversity and reservoir, absence of viral evolution [4] make these extraordinary subjects an exceptional model of persistent viral remission, that overlap largely with the described exceptional elite controllers [31, 32, 33] or even with a model of HIV eradication. In fact, the provocative hypothesis of whether some of these subjects have been able to eradicate HIV needs to be proven.

We encountered several limitations while performing this work such as the low number of HLA‐B*57 typed patients, responsible for the loss of statistical power in multivariate analysis and that we were unable to include data on IL28B genotype and liver fibrosis. However, this is the largest HIV‐controller cohort analysed in relation to HCV exposition profile and that differentiates between persistent and transient HIV‐controllers that have allowed us to analyse the association of spontaneous HCV clearance with persistent HIV‐controller status. These analyses were not possible in other published HIV‐controller cohorts.

## 5 CONCLUSIONS

This study shows an association between spontaneous persistent HIV‐control and HCV spontaneous clearance. The study findings support the idea of preserved immune mechanisms in persistent HIV control implicated in HCV spontaneous clearance. These results highlight persistent HIV‐controllers but not transient controllers as a good model of functional HIV cure.

## COMPETING INTEREST

The authors declare no conflicts of interest exist.

## AUTHORS’ CONTRIBUTIONS

ER‐M and ML designed and conceived the study. BD‐M and ER‐M performed the data analyses. BD‐M and ER‐M wrote the manuscript. LT‐D coordinated the management of the data from ECRIS cohort. PV, LL‐C, YM‐G, MG and ML contributed with the data management of the comparison cohort. MG, SR, CR, JG, L‐GC, RN, BJM, PM, CC, LA, FG, JDR, PV, LL‐C and ML contributed with patient’s supervision and data collection. All the authors critically reviewed, edited and approved the final manuscript.

## Supporting information


**Annex S1.** Clinical Centers and research groups which contribute to ECRIS.
